# Apoptosis-induced effects of extract from *Artemisia annua* Linné by modulating PTEN/p53/PDK1/Akt/ signal pathways through PTEN/p53-independent manner in HCT116 colon cancer cells

**DOI:** 10.1186/s12906-017-1702-7

**Published:** 2017-04-28

**Authors:** Eun Ji Kim, Guen Tae Kim, Bo Min Kim, Eun Gyeong Lim, Sang-Yong Kim, Young Min Kim

**Affiliations:** 1 Department of Biological Science and Biotechnology, College of Life Science and Nano Technology, Hannam University, 1646 Yuseong-daero, Yuseong-gu, Daejeon, 34054 Korea; 2 Department of Food Science & Bio Technology, Shinansan University, Daehakro Danwon-gu, Ansan-city, Gyeonggi-do Korea

**Keywords:** Phosphatase and tensin homolog (PTEN), p53-independent manner, *Artemisia annua* Linné, Apoptosis, HCT116 colon cancer cell

## Abstract

**Background:**

The extracts from *Artemisia annua Linné* (AAE) has been known to possess various functions including anti-bacterial, anti-virus and anti-oxidant effects. However, the mechanism of those effects of AAE is not well known. Pursuantly, we determined the apoptotic effects of extract of AAE in HCT116 cell. In this study, we suggested that AAE may exert cancer cell apoptosis through PTEN/PDK1/Akt/p53signal pathway and mitochondria-mediated apoptotic proteins.

**Methods:**

We measured 3-(4,5-dimethylthiazol-2-yl)-2,5-diphenyltetrazolium bromide (MTT) assay, lactate dehydrogenase (LDH) assay, Hoechst 33342 staining, Annexin V-PI staining, Mitopotential assay, immunofluorescence (IF) and Western blotting. Accordingly, our study showed that AAE treatment to HCT116 cells resulted in inhibition of PDK1, Akt, MDM2, Bcl-2, and pro-caspase 3 as well as activation of PTEN, p53-upregulated modulator of apoptosis (PUMA), Bax and Bak expression. Also we measured in vivo assay that xenograft model, H&E assay, TUNEL assay and IHC.

**Results:**

AAE induced apoptosis via PTEN/p53/PDK1/Akt signal pathways through PTEN/p53-independent manner. AAE inhibit cell viability and increase LDH release in HCT116 colon cancer cell. Also, AAE increase apoptotic bodies, caspase −3,7 activation and reduces mitochondria membrane potential. AAE regulates cytochrome c translocation to the cytoplasm and Bax translocation to the mitochondrial membrane in an Immunofluorescence staining and increase PTEN and p53 expression in an in vivo tumor xenograft model. To elucidate the role of the PTEN/p53/PDK1/Akt signal pathways in cancer control, we conditionally inactivated PTEN/p53/PDK1/Akt signal pathways. We used inhibitors of PTEN, p53, PDK1, Akt. In consequence, these results indicate that AAE induced apoptosis by means of a mitochondrial event through the regulation of proteins such as Bax, Bak and cytochrome *c* in PDK1/Akt signaling pathways via PTEM/p53-independent manner.

**Conclusions:**

We confirmed the apoptotic effect of extracts of AAE by Modulating PTEN/p53/PDK1/Akt/Signal Pathways through PTEN/p53-independent pathwaysin HCT116 colon cancer cell.

## Background

*Artemisia annua* Linné is an annual plant that is chrysanthemum family. This plant is primarily found in the tropical zones of Asia along streets and in fields. Since ancient times, *Artemisia annua* Linné has been used as an antipyretic, hemostatic, as a treatment for skin diseases, and an insecticide. In addition, its antibacterial, antiviral and antioxidant properties allow it has been used as a traditional herbalmedicine [1]. This plant also contains various bioactive compounds [2–5].

The antioxidant activity of phenolic compounds in *Artemisia annua* Linné has been reported [6]. Artemisinin, the main element of sweet wormwood, is being used for medical uses, such as anti-malarial activity [7–9]. In previous studies, they determined that when using *Artemisia annua* Linné on extracts from breast cancer, cervical carcinoma cells, stomach cancer, and for cell growth inhibitory effect that there is a cancer cell [10, 11]. Furthermore, selective necrosis of breast cancer cells was proven to be anti-cancer active. This brought attention to the world to consider taking action with herbal remedies [12]. Some studies have reported the effect of *Artemisia annua* Linné added to food and feed. However, the mechanism of the effects of *Artemisia annua* Linné is not well known [13].

PTEN (Phosphatase and TENsin homolog deleted on chromosome ten), a tumor-suppressorgene with dual lipid and protein phosphatase activity, antagonizes the PI3K/AKT signaling pathway and suppresses cell survival, as well as cell proliferation. Also, PTEN can inhibit the activation of Akt. This effect has been attributed to PTEN reducing the availability of Phosphatidylinositol (3,4,5)-trisphosphate (PIP3; 2–3) [14–16]. The serine/threonine kinase Akt is phosphorylated and activated by PDK1 (phosphoinositide-dependent protein kinase-1) [17].

PDK1 activation phosphorylates Akt at thr308. Once phosphorylated in T308, phosphorylation additionally occurs at S473by PDK2 [18]. Akt activation induces different cell survival mechanisms [19]. Akt plays a central role in many cellular processes that establish survival factor and exert anti-apoptotic activation. Also, Akt activation induces cell cycle progression [20]. In another case, Akt prevented apoptosis via phosphorylation and translocation of MDM2 (Murine double minute 2) into the nucleus [21, 22]. MDM2 interacts with p53 and inhibits it. Under normal circumstances, p53 is maintained at very low levels by ubiquitination and degradation [23]. The p53 gene, a tumor suppressor, plays a key role in the induction of apoptosis and cell cycle arrest in response to a variety of stress genes, including the blocker of cellular DNA damage repair [24]. p53 is a nuclear DNA-binding phosphor-protein. It is a transcriptional activator that can exert transcriptional repression of specific targeted genes [25]. Also, p53 interacts directly with cell proliferation-mediated proteins. The direct interaction of p53 activates apoptotic proteins into mitochondrial outer membrane permeabilization (MOMP) [26].

Mitochondria are well known for playing a key role in activating apoptosis. The mitochondrial apoptosis pathway is mediated via Bcl-2 family proteins [27, 28]. Bcl-2 family proteins are divided into anti-apoptotic proteins such as Bcl-XL, Bcl-w, Mcl-1 and pro-apoptotic proteins such as Bax, Bak and Bok. In normal cells, Bax exists as a monomer in the cytosol and translocates to mitochondria, experiencing conformational changes to form oligomers during apoptosis. On the other hand, Bak resides on mitochondria. During apoptosis, Bak changes to form oligomers identical to Bax. The activation of Bax and Bak regulates cytochrome c release to cytosol from the mitochondria via alteration of MOMP [29, 30]. Released cytochrome c induces apoptosis by activating last effectors caspase (caspase-3/−7) [31].

In this study, we investigated the effects of *Artemisia annua* Linné extract (AAE) on apoptosis in HCT116colon cancer cells. We suggested that AAE induced apoptosis through PTEN/PDK1/Akt/p53signal pathways and mitochondria-mediated apoptotic proteins.

## Methods

### Reagents and chemicals

AAE was purchased from Daejeon Oriental Herbal Market (Deojun, Korea).3-(4,5-Dimethylthiazol-2-yl)-2,5-Diphenyltetrazolium Bromide (MTT) was purchased from Sigma-Aldrich (St. Louis, MO, USA). The Pierce lactate dehydrogenase (LDH) Cytotoxicity Assay kit was purchased from Thermo Fisher Scientific (Waltham, MA, USA). The Muse Annexin V and Dead Cell Assay Kit, The Muse Caspase-3/7 Kit and the Muse MitoPotential Kit were purchased from Millipore (Darmstadt, Germany). MitoTracker was purchased from Molecular Probes (Eugene, OR, USA). Specific antibodies that recognized phosphorylated (p)Akt (Ser473) (4060S), (p)Akt (Tre308) (3038S), (p)PTEN (9549P), (p)PDK1 (3430P), PUMA (4976P) Bax (5023P), Bak (6947), pro-caspase-3 (9665), Bcl-2 (2876) and β-actin (4967) total formed (t)Akt (4060P), (t)PDK1 (3062P) were obtained from Cell Signaling Technology (Beverly, MA, USA). The total formed (t)PTEN (SC-7974) was purchased from Santa Cruz Biotechnology (Dallas, TX, USA), (t)MDM2 (NBPI-02158SS) was purchased from Novus Biologicals (Littleton, CO, USA) and (p)MDM2 was purchased from Abcam (Cambridge, MA, USA). LY294002 (PI3K/Akt inhibitor), Pifithrin-α (p53 inhibitor) were purchased from Calbiochem (San Diego, CA, USA), Nutlin-3 (MDM2 inhibitor) and BX-795 (PDK1 inhibitor) were purchased from Sigma-Aldrich (St Louis, MO, USA), BpV (PTEN inhibitor) was purchased from Santa Cruz Biotechnology (Dallas, TX, USA). Horseradish peroxidase (HRP)-conjugated Goat Anti-Mouse (PA1–30126) and Goat Anti-Rabbit (166–2408) secondary antibodies were purchased from Thermo Fisher Scientific, Inc., and Bio-Rad Laboratories, Inc., (Tokyo, Japan), respectively.

### Preparation of *Artemisia annua Linné* extract

100 g of the powdered AAE was extracted with 800 mL of 95% EthOH for 72 h. The extract was filtered through qualitative filter paper no. 1 (Toyo Roshi Kaisha, Ltd.; Tokyo, Japan) and concentrated with a rotary evaporator to remove the ethanol. AAE was dissolved in dimethyl sulfoxide (DMSO) prior to treatment and stored at −20 °C. The final concentration of AAE in the culture medium was controlled at 30-60 μg/ml.

### Cell culture

HCT116 cells were obtained from the American Type Culture Collection (ATCC; Rockville, MD, USA). The cells were grown in RPMI-1640 medium (Hyclone Laboratories Inc.)containing 10% fetal bovine serum (FBS) and 1% antibiotics at 37 °C in a 5% CO_2_ incubator. The cells were sub-cultured by detachment with Trypsin-EDTA (Hyclone Laboratories Inc.)and re-seeded at 1 × 10^6^ cells/mL per 100 mm plate every 48 h.

### 3-(4,5-Dimethylthiazol-2-yl)-2,5-diphenyltetrazolium bromide (MTT) assay

HCT116 cells and fibroblast cells (1 × 10^5^) were seeded onto 12-well plates and treated with AAE at concentrations of 30, 40, and 60 μg/mL for 24 h. Certain samples were pre-treated with a respective inhibitor (20 μMLY294002, 20 μM Nutlin, 20 μM pifithrin-α, 1 μM BpV and 5μMBX-)for 30 min prior to treatment with AAE. The selective medium removed and then incubated with20μl of MTT solution (5 mg/ml MTT in PBS) for 1 h. Converted purple formazan dye from MTT was solubilized in DMSO and optical densities were measured at 595 nm.

### Lactate dehydrogenase (LDH)assay

Cells were seeded at 2.5 × 10^5^ cells/mL per well in a 96-well plate and incubated for 24 h. The cells were then treated with AAE (30, 40, and 60 μg/mL) and incubated at 37 °C in a 5% CO_2_ atmosphere. After 24 h, the high control cells (maximum LDH release) were treated with cell lysis solution from the LDH Cytotoxicity Assay Kit for 30 min. The absorbance of the solution in each well was determined using a microplate reader (Bio-Rad Laboratories, Inc.) at 490 and 655 nm.

### Cells morphology

Cells were seeded at 1 × 10^5^ cells/mL in 6wellplatesandtreatedwithAAEfora 24 h time period at 30, 40, and 60 μg/ml concentrations.

### Hoechst 33342 staining

Cells were seeded at 1 × 10^4^ cells/mL in a 12-well plate with cover glasses and incubated for 24 h. Following incubation, the cells were treated with the AAE (30, 40, and 60 μg/mL) for 24 h at 37 °C in a 5% CO_2_ atmosphere. Cells were stained with Hoechst33342 for 30 min. Slides were washed with PBS and mounting fluid was poured over them. The slides were covered with a cover slip and sealed with nail polish. Fluorescence was measured by using a fluorescence microscope (Carl Zeiss, Germany).

### Cell apoptosis assay

HCT116 cell apoptosis was assayed using the Muse™ Annexin V and Dead Cell Kit (Merck Millipore, Guyancourt, France) according to the user’s guide. A total of 1x10^5^cells were collected by centrifugation (3000 rpm, 5 min) and washed with PBS. Cells were resuspended in a RPMI-medium with 1% bovine serum albumin and 10% FBS, mixed with the Muse™ Annexin V and Dead Cell reagent, and then incubated for 20 min at room temperature in the dark. Assay results were measured using the Muse™ Cell Analyzer.

### Caspase-3/7 activity analysis

Cells were seeded at 1 × 10^5^ cells/mL per plate in a 6-well plate and incubated for 24 h. Following incubation, the cells were treated with AAE (30, 40, and 60 μg/mL) for 24 h at 37 °C in a 5% CO_2_ atmosphere. HCT116 cell caspase activity was assayed using the Muse Caspase 3/7 Assay Kit according to the user’s guide. A total of 1 x 10^5^cells were collected by centrifugation (3000 rpm, 5 min) and washed with PBS. Cells were resuspended in a 1X Assay Buffer BA, mixed with the Muse™ Caspase-3/7 reagent, and then incubated for 20 min at 37 °C in a 5% CO_2_ atmosphere in the dark. After incubation, 150 μL of Muse™ Caspase 7-AAD working solution was added to each tube. The solution was mixed thoroughly by pipetting up and down, also know as vortexing, at a medium speed for 3 to 5 s. It was then incubated at room temperature for 5 min in the dark. Assay results were measured using the Muse™ Cell Analyzer.

### Western blotting

Cells were seeded at 1 × 10^5^ cells/mL per plate in a 6-well plate and incubated for 24 h. The cells were then treated with AAE (30, 40, and 60 μg/mL) and incubated at 37 °C in a 5% CO_2_ atmosphere. Certain samples were pre-treated with the respective inhibitor (20 μMLY294002, 20 μM Nutlin, 20 μM pifithrin-α, 1 μM Bp V and 5 μMBX-795) for 30 min prior to treatment with AAE. Cells were rinsed twice with ice cold PBS and scraped with a lysis buffer (50 mM Tris-HCl pH 8.0, 150 mMNaCl, 1% NP40, 0.5% sodium deoxycholate, 1 mM PMSF) and subjected to western blot analysis. The primary antibody was allowed to react overnight at 4 °C and the second antibody reacted for 90 min at room temperature with gentle agitation. Following washing the samples four times with 1X Tris Buffered Saline with Tween 20 (TBST) for 10 min at room temperature, proteins were detected using Super Signal West Pico Chemiluminescent Substrate (PI34080; Thermo Fisher Scientific, Inc., Waltham, MA, USA) and visualized on CP-BU new X-ray film (Agfa HealthCare, Inc., Mortsel, Belgium).

### Mitochondrial membrane potential assay

Cells were seeded at 1 × 10^5^ cells/mL per plate in a 6-well plate and incubated for 24 h. Following incubation, the cells were treated with the AAE (30, 40, and 60 μg/mL) for 24 h at 37 °C in a 5% CO_2_ atmosphere. HCT116 cell caspase activity was assayed using the Muse MitoPotential Kit according to the user’s guide. A total of 1 x 10^5^cells were collected by centrifugation (3000 rpm, 5 min) and washed with PBS. The supernatant was then removed and the cell pellets were stained with the Muse MitoPotential Kit (Merck Millipore, Guyancourt, France) for 25 min at 37 °C.The data was analyzed using the Muse™ Cell Analyzer Assay.

### Fraction of mitochondria and cytosol proteins

We used a Mitochondria/Cytosol Fraction Kit (Abcam, Cambridge, MA, USA). Cells were seeded at 1 × 10^6^/ml on a 100 mm plate and incubated for24 h. After incubation, cells were treated with AAE for 24 h at 37 °C in a 5% CO_2_ atmosphere. Cells were harvested by trypsinization, collected by centrifugation, washed with PBS, and homogenized in an ice cold cytosol extraction buffer mix containing DTT and protease inhibitor using a sonicator. The homogenates were centrifuged at 3000 rpm for 10 min at 4 °C and the supernatants were collected. The supernatants were centrifuged at 13000 rpm for 30 min at 4 °C and collected. The supernatant cytosol proteins and pellets were resuspended with ice cold mitochondria extraction buffer containing DTT and a protease inhibitor for mitochondria proteins.

### Immunofluorescence (IF)staining

Cells were seeded at 1 × 10^4^ cells/mL in a 12-well plate with cover glasses and incubated for 24 h. The cells were treated with the AAE (30, 40, and 60 μg/mL) for 24 h at 37 °C in a 5% CO_2_ atmosphere. The cells were stained with MitoTracker**.** for 30 min at 37 °C in a 5% CO_2_ atmosphere. Cells were fixed with 3.7% formaldehyde for 20 min and permeabilized with 0.2% Triton X-100 for 20 min. Cells were washed with PBS twice and reacted with cytochrome c*,* Bax and Bak antibodies overnight at 4 °C. Cells were washed with PBS twice and reacted with a secondary antibody for 1 h 30 min. Fluorescence was detected by confocal microscopy (Olympus; Tokyo, Japan).

### Xenograft model

Five-week-old male Balb/c nudemice were obtained from SLC (SLC; Tokyo, Japan) Five mice made up the control group while five other mice made up the experimental and delivery groups for each concentration. For tumor induction, HCT116 human colon cancer cells (2.5 × 105 cells/0.1 ml) were subcutaneously injected into the left flank of the mice (each group had 10 animals). One week after the injection of cells, the mice were co-treated with AAE20, 40 mg/kg/day and 0.2cm^3^ PBS/DMSO for 21 days. Tumor size was measured by taking two perpendicular diameter measurements, using a caliper, every 2 days. The tumor volume was calculated using the following formula: V = 1/2 (length x width). The body weight of each animal was measured at a set time, once per week. After the 3-week treatment, the tumor was removed and frozen in liquid nitrogen for western blot analysis or fixed with formalin for immunohistochemistry, TUNEL and H&E staining. All of the animal experiments were approved by the Ethics Committee for Animal Experimentation of Hannam University (Daejeon, Korea, HNU 2016–9).

### TUNEL assay

Levels of apoptosis in distal colon tissue were determined using the TdT-mediated dUTP nickend labeling (TUNEL)method. Tumor specimens from mice were fixed in 10% formaldehyde, embedded in paraffin and sectioned into 5 μm thick slices. Tissue sections were processed according to manufacturer’s instructions for the Apop Tag Peroxidase In Situ Apoptosis Detection Kit (Vector Laboratories; Burlingame, CA, USA).

### Immunohistochemistry

Tumor specimens from mice were fixed in 10% formaldehyde, embedded in paraffin and sectioned into 5 μm thick slices. Consecutive thin cryosections (5 μm) of OCT compound (Sakura Finetek; Torrance, CA,USA) embedded tumor tissues were fixed in acetone at 4 °C for 10 min. After washing in PBS, the sections were treated with3% H_2_O_2_ for 10 min to block endogenous peroxidase activity. The sections were then blocked with normal rabbit serum. Last, the sections were blocked and washed in PBS and incubated with specific antibodies overnight at 4 °C. Negative controls were incubated with the primary normal serum IgG for the species from which the primary antibody was obtained.

### Statistical analysis

Cell viability was statistically analyzed using unpaired SPSS Student’s ANOVA-tests and t-tests (SPSSChicago, IL, USA). *p* < 0.05 was considered statistically significant.

## Results

### AAE reduces cell proliferation in HCT116 colon cancer cells

**Fig. 1:**
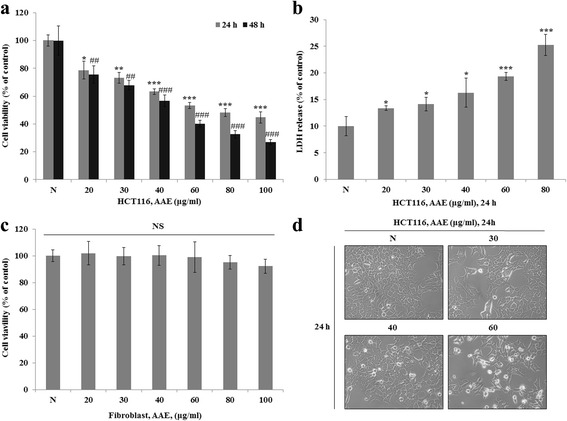
AAE reduces cell proliferation in HCT116 colon cancer cells. **a**, **c** Cell viability was measured by MTT assay. **a** is HCT116 cell line. **c** is Fibroblast cell line. The statistical analysis of the data was carried out by use of an T-test. */#p < 0.05, **/##'p < 0.01 and ***/###p < 0.001 (each experiment, *n* = 3). **b** LDH assay was performed for assessing cell deaths. Cytotoxicity was induced by AAE. The statistical analysis of the data was carried out by use of an T-test. *p < 0.05, **p < 0.01 and ***p < 0.001 (each experiment, *n* = 3). **d** AAE affects the morphology of HCT116 cells, and promotes cell death in a dose-dependent manner. HCT116 were treated with AAE (0, 30, 40, and 60 ìg/ml) for 24 h

We investigated the cytotoxic effects of AAE through the use of a MTT Assay and LDH Release Assay on HCT116 colon cancer cells. Also, we determined the cytotoxic effects of AAE on normal human fibroblast cells. We treated cells with AAE (20–100 μg/ml) for 12 or 24 h, and then assayed using a MTT Assay and LDH Assay, creating a cell morphology image used to investigate cellular viability. Figure 1a showed a decrease in cell viability. As shown in Fig. 1b, LDH release significantly increased following treatment with 20, 30, 40, 60, 80, and 100 μg/ml of AAE. However, AAE had no effect on cellular viability in normal human fibroblast cells (Fig. 1c). Compared with the control group, the group treated with AAE had induced typical apoptotic cell morphology in HCT116 cells (Fig. 1d).

### AAE induces apoptosis and regulates pro-apoptotic proteins in HCT116 colon cancer cells

**Fig. 2:**
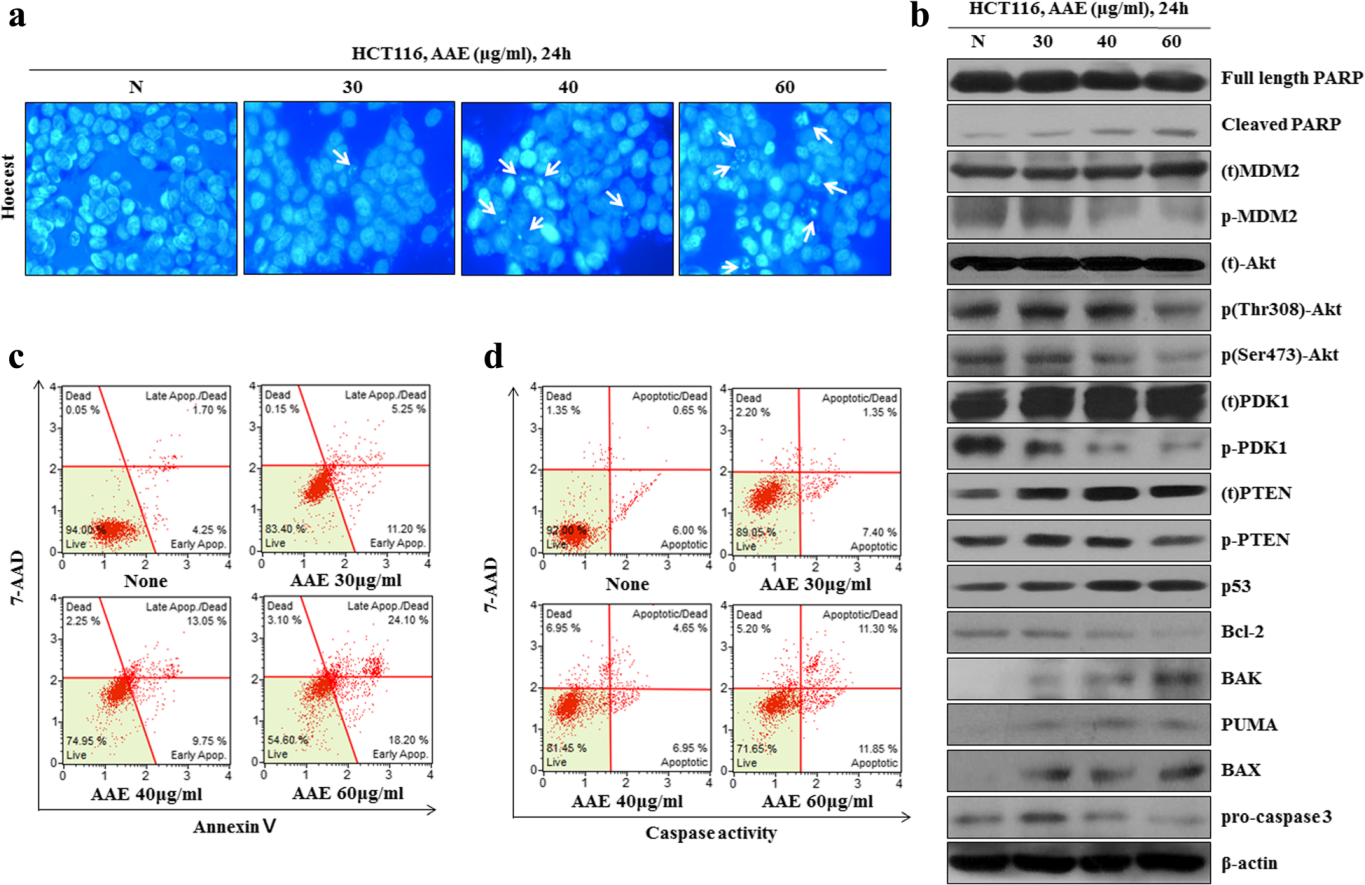
AAE induces apoptosis and regulates pro-apoptotic proteins in HCT116 colon cancer cells. **a** Cell apoptosis observed using Hoechst 33342 staining. HCT116 were treated with AAE (0, 30, 40, and 60 ìg/ml) for 24 h. Fluorescence was detected using a fluorescence microscope. *Arrows* indicate apoptotic bodies, which were DNA fragments produced when apoptosis occurred. **b** Apoptotic effects of different concentration AAE were evaluated by Muse™ Annexin V and Dead Cell Assay Kit. HCT116 were treated with AAE (0, 30, 40, and 60 ìg/ml) for 24 h. Data analyzed by flow cytometry. **c** HCT116 were treated with 0, 30, 40, and 60 ìg/ml of AAE 24 h, caspase 3/7 activity was analyzed using a Muse™ Caspase-3/7 kit, as described in Materials and Methods. **d** Cells were treated with the indicated concentrations of AAE for 24 h. The expression of PTEN, PDK1, Akt (Thr308 and Ser473), MDM2, p53, PUMA,Bcl-2, pro-caspase3 and the activation of Bax, Bak and cleaved PARP were analyzed by western blot analysis

To determine whether AAE induction decreases cell viability involved cell death, we exerted a staining procedure using the Hoechst 33342 dye and the Muse™ Annexin V and Dead Cell Kit. Figure 2a shows that treatment with AAE (30–60 μg/ml) for 24 h, leads to the apoptotic bodies increasing in a dose-dependent manner. As shown in Fig. 2b, the ratio of Annexin V-positive cells was low in the control group, while the percent of Annexin V-positive cells increased in the AAE treatment groups.

In order to determine the influence of AAE on caspase-3 activation, a caspase-3 activity assay was performed using a Muse Caspase 3/7 Activity Assay. HCT116 colon cancer cells were treated with AAE at 30, 40, and 60 μg/mL for 24 h. In Fig. 2c, the levels of caspase-3 increased in a dose-dependent manner in each cell line. Changes of PTEN, p-PDK1, p-Akt, p-MDM2, p53, Bcl-2 and apoptosis-related proteins such as Bak, Bax, PUMA and pro-caspase-3 after treatment with AAE were determined by western blot analysis. The results showed that an increased concentration of AAE increased the reduction of cell survival proteins such as p-PDK1, p-Akt, p-MDM2, Bcl-2and pro-caspase-3. Moreover, the levels of PTEN, p53 and the mitochondria-mediated apoptotic proteins Bak, Bax and PUMA increased in a dose-dependent manner (Fig. 2d).

### AAE reduces the mitochondrial membrane potential

**Fig. 3:**
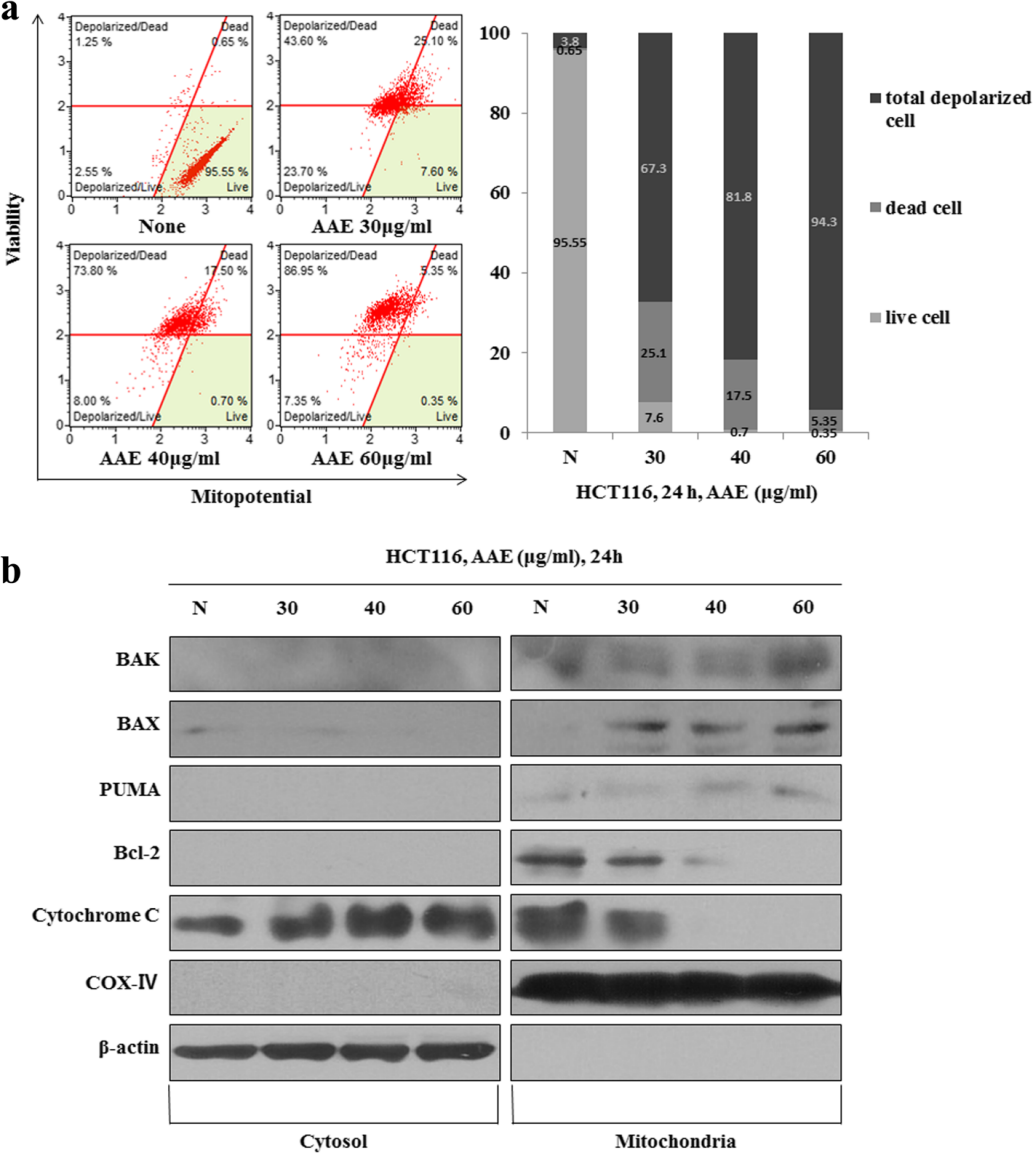
AAE reduces the mitochondrial membrane potential. **a** Mitochondria membrane potential were evaluated by Muse™ Mitopotential kit. Cells were treated with different concentration of AAE (HCT116 were treated with 0, 30, 40, and 60 ìg/ml of AAE) for 24 h. **b** Fraction of mitochondria/cytosol protein levels were analyzed by Western blotting

To investigate the mechanism of AAE-induced apoptosis, we employed a staining procedure using with the Muse MitoPotential Kit. After treatment with AAE (30-60 μg/mL) for 24 h, the mitochondrial membrane potential reduced in a dose-dependent manner (Fig. 3a). In order to examine whether AAE-induced apoptotic cell death correlates with mitochondrial membrane potential, the levels of these proteins were observed in cytosol as well as mitochondria fractions. In Fig. 3b, the levels of pro-apoptotic mitochondrial proteins increased and anti-apoptotic mitochondrial proteins decreased in the mitochondrial fraction.

### AAE regulates cytochrome c translocation to the cytoplasm and Bax translocation to the mitochondrial membrane

**Fig. 4:**
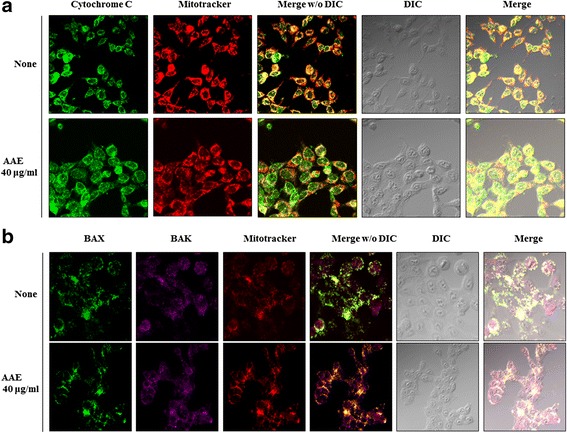
AAE regulates cytochrome c translocation to the cytoplasm and Bax translocation to the mitochondrial membrane. **a** AAE regulated cytochrome C translocation from mitochondria to cytosol. Also, (**b**) AAE induced BAX activation from mitochondria outer membrane to cytosol and BAX/BAK oligomerization. Cell were treated with AAE (40ìg/ml) for 24 h. Target protein and mitochondria were stained with fluorescence lag and cell locations were indicated using a DIC image

These results showed that AAE induced cytochrome c release from the mitochondria to the cytosol and Bax translocation from the cytosol to the mitochondria. We treated AAE of 40 μg/mL for 24 h and stained cells in order to visualize the mitochondria, cytochrome c, Bax and Bak (Fig. 4).

### AAE-induced apoptosis by p53-independent manner

**Fig. 5:**
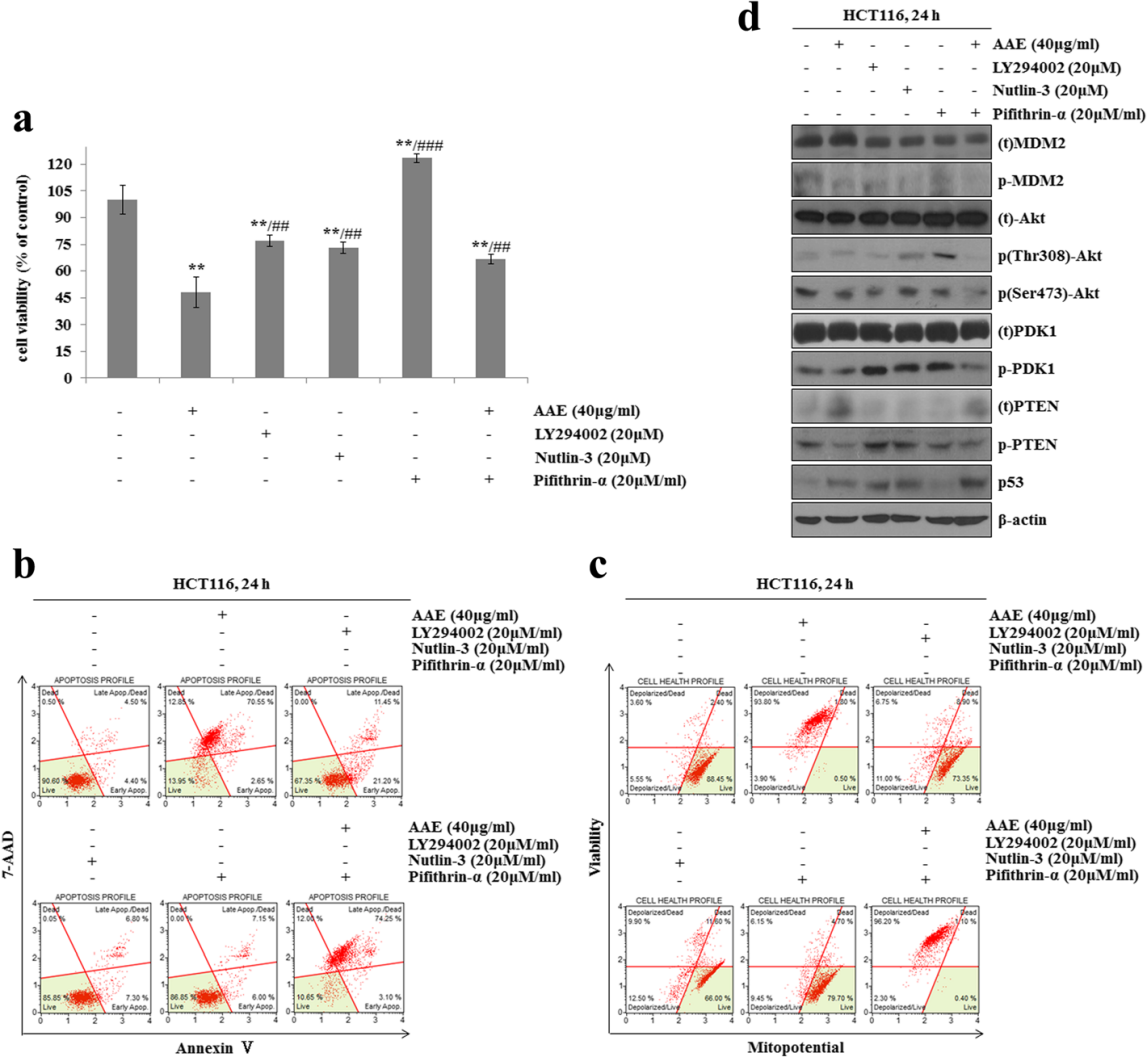
AAE-induced apoptosis by p53-independent manner. Cells were treated with 20 μM LY294002, 20 μM Nutlin-3, 20 μM Pifithrin-á and 40 μg/ml AAE for 24 h. **a** Cells viability was measured by MTT assay (20 μM LY294002, 20 μM Nutlin-3, 20 μM Pifithrin-á 40 μg/ml AAE). The statistical analysis of the data was carried out by use of an an T-test. */#p < 0.05, **/##p < 0.01 and ***/###p < 0.001 (each experiment, *n* = 3). **b** Apoptotic effects of different concentration AAE were evaluated by Muse™ Annexin V and Dead Cell Assay Kit. HCT116 were treated with 20 μM LY294002, 20 μM Nutlin-3, 20 μM Pifithrin-á 40 μg/ml AAE for 24 h. **c** Mitochondria membrane potential were evaluated by Muse™ Mitopotential kit. HCT116 were treated with 20 μM LY294002, 20 μM Nutlin-3, 20 μM Pifithrin-á 40 μg/ml AAE for 24 h. **d** Cells were treated with 20 μM LY294002, 20 μM Nutlin-3, 20 μM Pifithrin-á and 40 μg/ml AAE for 6 h. The expression of PTEN, PDK1, Akt (Thr308 and Ser473), MDM2, p53 were analyzed by western blot analysis

We examined the association between AAE-induced apoptosis and p53. First, we progressed a MTT assay, Annexin V and Dead cell staining, MitoPotential analysis and western blot after AAE treatment of 20 μM LY294002, 20 μM Nutlin, and 20 μM Pifithrin-α, along with20 μM Pifithrin-α prior to AAE, in HCT116 cells. Figure 5a showed that the group treated with Nutlin, Pifithrin-α only indicated similar cell viability to the control group, while treatment with AAE, LY294002 and the AAE co-treated Pifithrin-α group increased cell viability. As shown in Fig. 5b, the ratio of Annexin V-positive cells was low in the control and treatment with Nutlin, Pifithrin-α only groups, however, the percent of Annexin V-positive cells increased in the treatment of LY294002, AAE and AAE co-treatment Pifithrin-α group. To examine the mechanism by association of AAE with p53, we used a Muse MitoPotential Kit. While treatment with AAE, 20 μM LY294002, 20 μM Nutlin, 20 μM Pifithrin-α and 20 μM Pifithrin-α prior to AAE incubated for 24 h, the mitochondrial membrane potential reduced in the LY294002, AAE and AAE co-treatment Pifithrin-α group however, treatment with the Nutlin and Pifithrin-α only groups indicated similar mitochondrial membrane potential to the control group (Fig. 5c).

Changes of PTEN, p-PDK1, p-Akt, p-MDM2 and p53 after treatment with AAE, 20 μM LY294002, 20 μM Nutlin, 20 μM Pifithrin-α and 20 μM Pifithrin-α co-treatment with AAE were determined by western blot analysis. The results showed that the levels of cell survival proteins such as p-PDK1, p-Akt, p-MDM2 decreased but levels of PTEN and p53 increased in the treatment of LY294002, AAE and AAE co-treatment Pifithrin-α group. However, treatment with the Nutlin, Pifithrin-α only group showed similar results of cell survival proteins levels to that of PTEN and p53, the control group (Fig. 5d).

### AAE induces apoptosis through regulation of intracellular signaling pathways in an HCT116 xenograft model

**Fig. 6:**
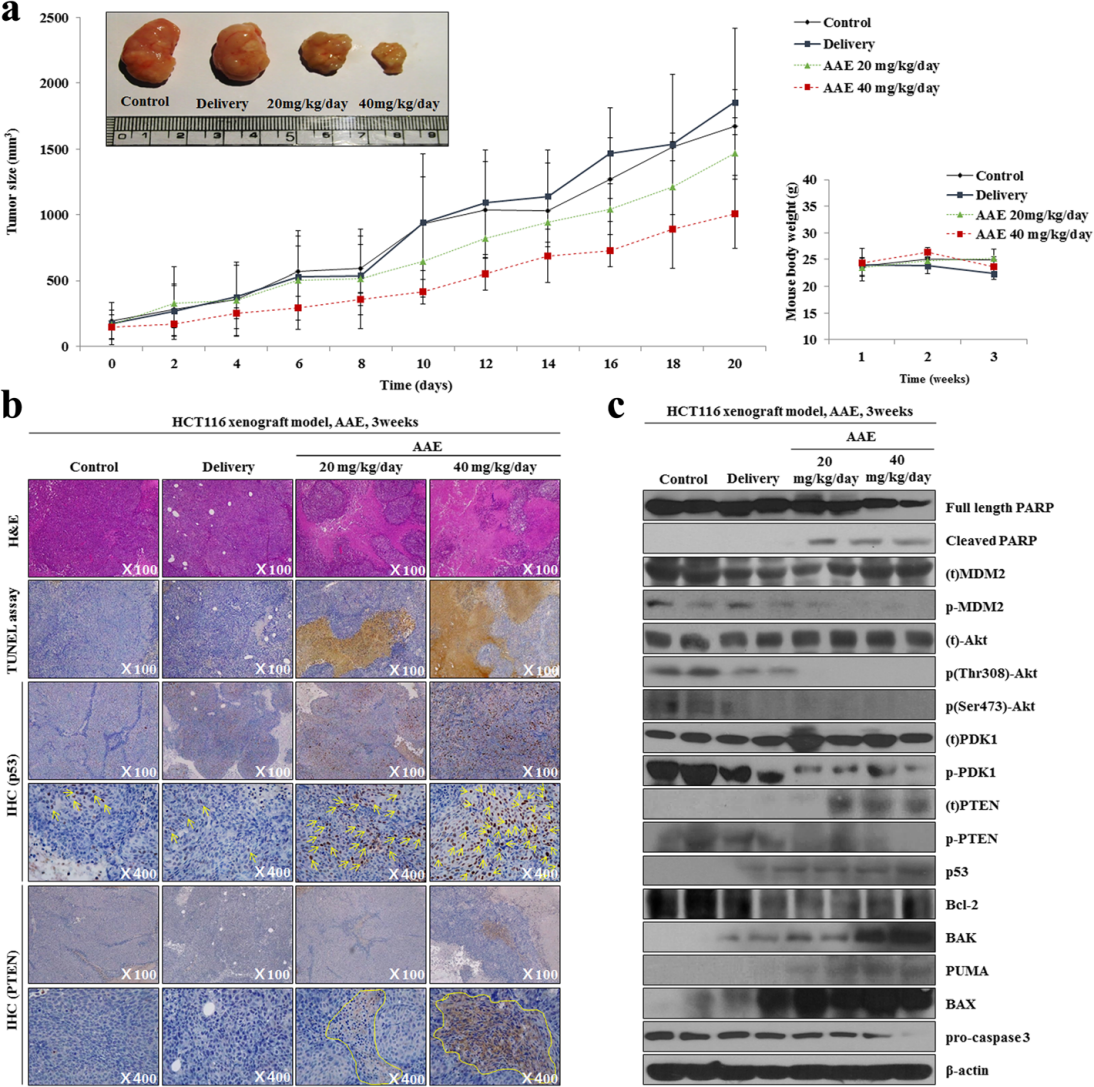
AAE induced apoptosis through regulated mitochondria signaling pathways in HCT116 Xenograft model. **a** Tumor size and body weight of HCT116 Xenograft model mouse. AAE direct inject to tumor and tumor size were redialed each 2 day and tumor image. **b** Histological experiments of tumor tissue. H&E staining, TUNEL assay and Immunohistochemical (IHC) assay were were performed at 21 day. The arrow and around indicate that specific proteins were activated by AAE treatment. **c** The expression of PTEN, PDK1, Akt (Thr308 and Ser473), MDM2, p53, PUMA,Bcl-2, pro-caspase3 and the activation of Bax, Bak and cleaved PARP were analyzed by western blot analysis

In order to analyze the results of AAE treatment in an HCT116 xenograft model of tumor growth, we transplanted HCT116 cells into mice and constructed a human colon cancer xenograft model. We executed histological analysis on control, delivery (DMSO mixed PBS) and AAE (20, 40 mg/kg/day) tumor tissue stained with H&E using the TUNEL assay. The tumor volume in AAE-treated groups was less than in the control and delivery group, however, the mice weighed the same in the control group, delivery group and AAE (20, 40 mg/kg/day) group (Fig. 6a). The cancer tissue was degraded and the number of TUNEL positive cells increased in the AAE-treated group. Immunohistochemistry (IHC) analyses exhibited that p53 and PTEN-positive cells were increased in the AAE-treated group as compared to the control group and delivery group (Fig. 6b). Furthermore, as shown in Fig. 5c, apoptosis-related proteins increased in similar in vitro western blot results.

### AAE induces apoptosis by regulating the phosphorylation of PDK1 and Akt through the PTEN/p53-independent pathway

**Fig. 7:**
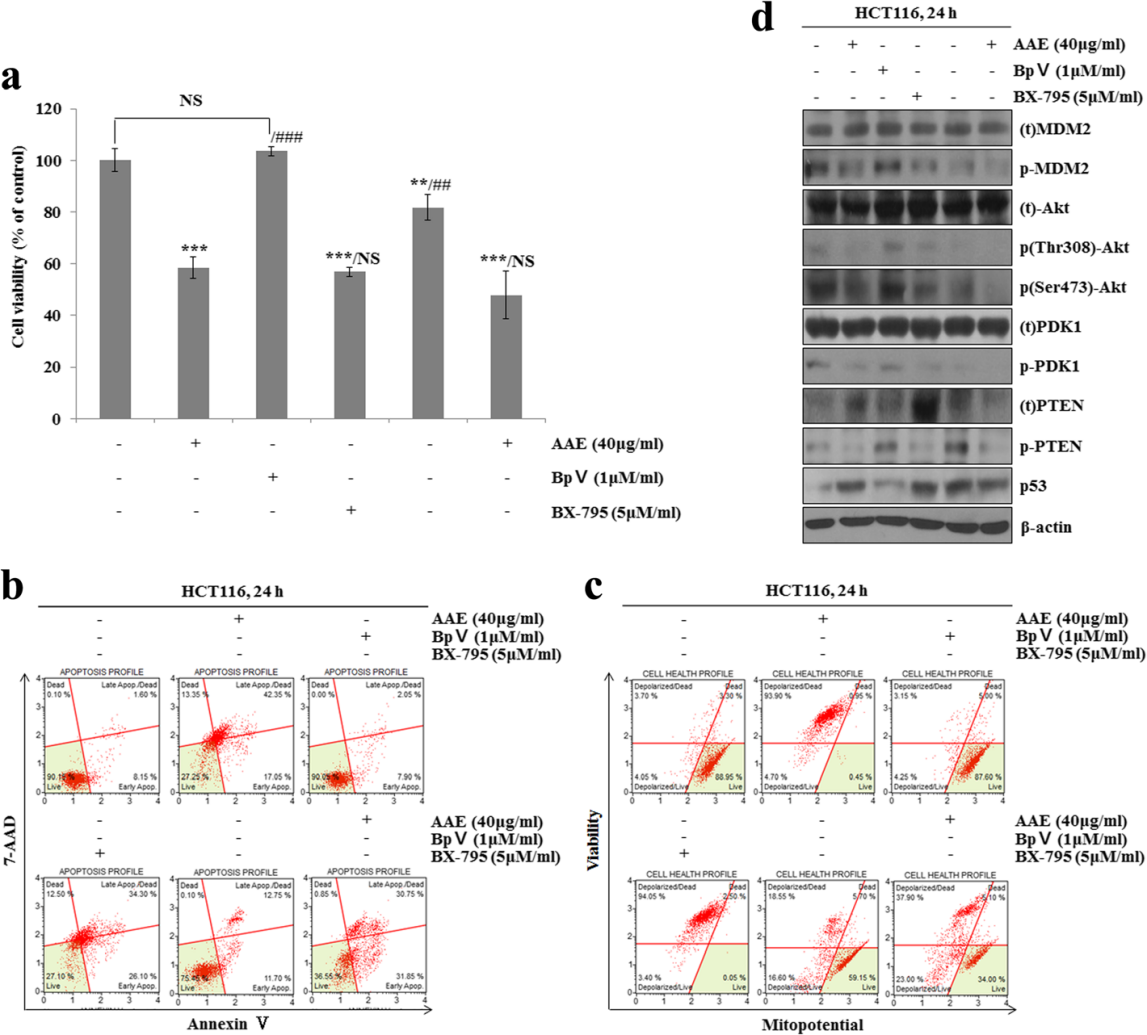
AAE induces apoptosis by regulating the phosphorylation of PDK1 and Akt through the PTEN/p53-independent pathway. Cells were treated with 1 μM BpV, 5 μM BX-795 and 40 μg/ml AAE for 24 h. **a** Cells viability was measured by MTT assay. The statistical analysis of the data was carried out by use of an an T-test. */#p < 0.05, **/##p < 0.01 and ***/###p < 0.001 (each experiment, *n* = 3). **b** Apoptotic effects of different concentration AAE were evaluated by Muse™ Annexin V and Dead Cell Assay Kit. HCT116 were treated with 1 μM BpV, 5 μM BX-795 and 40 μg/ml AAE for 24 h. **c** Mitochondria membrane potential were evaluated by Muse™ Mitopotential kit. HCT116 were treated with 1 μM BpV, 5 μM BX-795 and 40 μg/ml AAE for 24 h. for 24 h. **d** Cells were treated with 1 μM BpV, 5 μM BX-795 and 40 μg/ml AAE for 24 h. for 6 h. The expression of PTEN, PDK1, Akt (Thr308 and Ser473), MDM2, p53 were analyzed by western blot analysis

To determine the direct regulating AAE proteins, we progressed a MTT assay, Annexin V and Dead cell staining, MitoPotential analysis, and western blot after being treated with AAE, 1 μM BpV, 5 μM BX-795 and combination of AAE in HCT116 cells. The group treated with 1 μM BpV, 5 μM BX-795only indicated similar cell viability to the control group, while group receiving treatment AAE and AAE co-treated1 μM BpV and 5 μM BX-795 saw increased the cell viability (Fig. 7a). Figure 7b shows that the ratio of Annexin V-positive cells was low in the control and treatment with 1 μM BpV, 5 μM BX-795only group. However, the ratio of Annexin V-positive cells increased in the treatment AAE and AAE co-treated1 μM BpV and 5 μM BX-795groups. We also used a Muse MitoPotential Kit. Mitochondrial membrane potential was reduced in the group receiving AAE, 1 μM BpV, 5 μM BX-795 and a combination of AAE. However, treatment with 1 μM BpV, 5 μM BX-795only group indicated similar mitochondrial membrane potential to the control group (Fig. 7c).

The levels of PTEN, p-PDK1, p-Akt, p-MDM2 and p53 were determined by western blot analysis. Figure 7d shows that the levels of cell survival proteins such as p-PDK1, p-Akt, p-MDM2 decreased but levels of PTEN and p53increased in the treatment of AAE,1 μM BpV, 5 μM BX-795 and combination of AAE group. However, the treatment with 1 μM BpV, 5 μM BX-795 only group showed similar levels of cell survival proteins PTEN and p53 to the control group.

## Discussion

From old times, *Artemisia annua* Linné has been known for its anti-cancer, anti-viral and anti-bacterial properties, but the mechanisms were unknown. In this study, we investigated the effects of apoptosis and signal pathway via extract from *Artemisia annua* Linné in HCT116 colon cancer cells.

In this study, we focused on the effects of AAE on the induction of apoptosis. First, in order to determine the influence of AAE on cell viability, we performed a MTT assay and LDH assay after treatment with AAE. We confirmed that there is a range of cell damage contributing to significant inhibition of cell proliferation depending on the treatment.

Apoptosis at the mitochondrial level is completely dependent on Bax and Bak, as deficiency in the genes encoding these two proteins renders cells resistant to apoptosis and concomitant release of cytochrome c through the outer mitochondrial membrane. Cytochrome c, as a pro-apoptotic protein, plays an important role in triggering programmed cell death [32]. The release of cytochrome c from mitochondria directly triggers caspase-3 activation through formation of the cytochrome c containing apoptosome complex [33]. To confirm the apoptosis mechanism, we performed a Hoechst staining, an Annexin V/Dead cell staining, caspase activity, western blot, MitoPotential staining, fraction western blot and IF staining. Taking all of the results together, AAE induced apoptotic cell death. This cell death is the AAE controlled cytochrome c released from mitochondria to cytoplasm by formation of Bax/Bak oligomeric complexes and led to translocation onto the mitochondria outer membrane. Released cytochrome c by AAE treatment was an induced caspase-3 activity and this activity caused the apoptotic cell death.

Former studies demonstrated special compound-induced apoptosis via a p53-independent manner in HCT116 cells [34]. Thus, in order to confirm the association of AAE-induced apoptosis and p53, we treated Pifithrin-α (p53 inhibitor) in HCT116 cells. Our results showed an AAE-induced apoptosis via a p53-independent manner, was in cells treated with only AAE.

Our in vivo results showed AAE-induced apoptosis in a mouse xenograft model. The ratio of tumor growth was reduced in the AAE-injected group compared to the control and delivery groups. Also, the AAE-injected group revealed a growth of pro-apoptosis proteins and p53 expressed in a p53-independent manner.

When AAE-induced apoptosis occurred by p53-independent pathway, we used BpV (PTEN inhibitor), and BX-795 (PDK1 inhibitor) to determine the directly regulating proteins and signal pathway by AAE. In the MTT assay, through Annexin V staining and MitoPotential staining, we confirmed that AAE-induced apoptotic cell death is mitochondria-mediated apoptotsis by PTEN independent, PDK1 dependent pathways. Through the western blot on equal terms, we detected that AAE-induced apoptosis induced activation of apoptosis related proteins by regulating the PDK1 directly.

## Conclusions

In conclusion, our data reveals that AAE exerts apoptotic influences in the in vitro, in vivo situations via modulation of PDK1/Akt signaling pathways and the mitochondrial apoptosis pathway through the regulation of proteins such as Bax, Bak and cytochrome c in a PTEM/p53-independent manner.

## Abbreviations

AAE: Extracts from *Artemisia annua Linné*
DMSO: Dissolved in dimethyl sulfoxide
FBS: Fetal bovine serum
IF: Immunofluorescence
IHC: Immunohistochemistry
LDH: Lactate dehydrogenase
MOMP: Mitochondrial outer membrane permeabilization
MTT: 3-(4,5-Dimethylthiazol-2-yl)-2,5-diphenyltetrazolium bromide
PDK1: Phosphoinositide-dependent protein kinase-1
PTEN: Phosphatase and tensin homolog
TUNEL: TdT-mediated dUTP nickend labeling
